# Impact of meteorological factors on the occurrence of acute aortic dissection in Fujian Province, China: a single-center seven-year retrospective study

**DOI:** 10.1186/s13019-020-01227-7

**Published:** 2020-07-20

**Authors:** Zeng-Rong Luo, Ling-Li Yu, Shu-Ting Huang, Liang-Wan Chen, Qiang Chen

**Affiliations:** Department of Cardiovascular Surgery, Union Hospital, Fujian Medical University, Fuzhou, China

**Keywords:** Meteorological factors, Aortic dissection, Temperature

## Abstract

**Background:**

The aim of this study was to investigate the correlation between meteorological factors and the occurrence of acute aortic dissection (AAD) in Fujian Province, China.

**Methods:**

The clinical data of 2004 patients diagnosed with AAD in our hospital and the relevant local meteorological data from January 2013 to November 2019 were retrospectively analyzed.

**Results:**

The incidence of AAD had a clear tendency toward concentration, and the corresponding peak in terms of the occurrence date was from January 13 to 14. The average minimum temperature, the average maximum temperature, and the average daily temperature differences on the “day with AAD” were significantly lower than those on the “day without AAD”. From 5 days to 3 days before AAD onset, the average daily temperature difference showed a downward trend, but statistical analysis showed that the average minimum, average maximum and average daily temperature differences were not significantly different from the values 5 days to 0 days before AAD onset.

**Conclusions:**

The incidence of AAD is related to the season and month. The lowest average temperature may increase the incidence of AAD in patients with complicated cardiovascular diseases.

## Introduction

Acute aortic dissection (AAD) is a medical emergency with the characteristics of sudden occurrence and rapid progression. The results of previous studies have shown that the incidence of aortic dissection is related to hypertension, atherosclerosis, aneurysms, Marfan syndrome, aortic valve deformities, and previous aortic surgery history, which are risk factors for aortic dissection [1, 2]. Congenital connective tissue defects and pregnancy are also associated with the onset of aortic dissection [3]. Studies have found that environmental factors have a significant effect on the incidence of cardiovascular events [4–6]. The occurrence of aortic aneurysm rupture, acute myocardial infarction, cerebrovascular accidents and so on is related to climate change, but there is little research on the relationship between the occurrence of aortic dissection and climate change, especially in terms of Chinese data [7–9]. Payman’s research stated that they could not reveal any dependence of atmospheric pressure, air temperature or the presence of a full moon on the incidence of different types of AAD [10]; however, the correlation between AAD and meteorological conditions and whether the incidence is concentrated is still controversial. We used data from patients with AAD treated in our hospital in the past 7 years, combined with the same period of meteorological data in Fuzhou district, and observed and discussed the possible correlation between meteorological factors and the occurrence of AAD.

## Materials and methods

### The research subjects

A retrospective study was conducted to analyze the relevant clinical data from patients with AAD admitted to our hospital from January 2013 to November 2019. The inclusion criteria were as follows: 1, age ≥ 18 years old; 2, the first disease onset location of the patient was in Fujian Province; and 3, the accurate onset time was calculated according to the clinical time and the time from the visit to our hospital. Patients were diagnosed with AAD when the time from onset to consultation was less than 14 days according to computed tomography angiography (CTA), magnetic resonance imaging (MRI) or transthoracic echocardiography [11]. The exclusion criteria were as follows: 1, congenital aortic malformation; 2, Marfan syndrome; 3, connective tissue disease and vasculitis; and 4, a recent history of major organ surgery and trauma. This study complies with medical ethics standards and was approved by our ethics committee.

### Clinical data collection

Patients with repeated admissions were collected for the first diagnosis of AAD. The following data were recorded: the patient’s age and sex; the dissection type; the presence of hypertension, diabetes, impaired glucose tolerance, coronary heart disease, COPD, smoking, alcohol use or other past medical history; the diagnosis; the hospitalization time; and the AAD onset time. The collection of data from medical records was completed by one researcher. Another researcher created a database with Excel software and inputted and verified the data.

### Meteorological data collection

The study area was a typical southern hemisphere temperate monsoon climate with four distinct seasons in Fujian Province. Meteorological data were provided by the Fujian Meteorological Bureau. Records of the daily minimum temperature, average minimum temperature, daily maximum temperature, average maximum temperature, daily temperature difference and average daily temperature difference were collected from January 2013 to November 2019, and records of daily air quality from January 2016 to November 2019 were also collected. All data collection was performed by a single person. A database of meteorological data was created with Excel software, and the data were inputted and verified by the same researcher.

### Statistical analysis

#### Analysis of the trend of the centralized distribution of AAD

The data regarding AAD onset time were entered into SPSS 19.0 software, and the circular distribution method was used to calculate and hypothesize the peak incidence of aortic dissection. The peak incidence was usually described as the average angle (α) if there was a trend in the concentration; this measure could be converted from the onset time to an angle, that is, 12 months (365 d) in 1 year to 360° and 0.9863° per day [12]. The monthly median was used as the group median and converted into angle αi, that is, the January-month median was 15.5°, the February-month median was 45.5° and so on, to calculate the 12-month monthly median. The incidence peak period was reversed by the alpha of the circular Von Mises distribution; the authenticity of the peak distribution was confirmed by the homogeneity test, that is, to infer whether alpha was statistically significant.

#### Comparative analysis between the groups

A comparison of meteorological conditions between “days with AAD” and “days without AAD” and changes in average minimum temperature from 5 days before the onset of AAD to the day of onset of AAD was performed using SPSS 19.0 software. The Kolmogorov-Smirnov method was used to test the normality of the measurement data. The measurement data with a normal distribution are expressed as the mean ± standard deviation (x ± s). The comparison between groups was performed by single-factor analysis of variance. The LSD test was used for pairwise comparisons when the variances were homogeneous, and the Tamhane T2 test was used when the variances were uneven. Nonnormally distributed measurement data are expressed as the median (quartile) [M (QL, QU)]; the nonparametric Kruskal-Wallis H test was used for comparison among multiple groups, and the Mann-Whitney U test was used for comparison between the two groups. Count data are expressed as rates and were analyzed using the χ2 test. *P* < 0.05 was considered statistically significant.

## Results

### General information

A total of 2004 cases of AAD were included in this study. The sites of onset were Fujian Province areas. There were 1160 cases of aortic dissection type A and 844 cases of aortic dissection type B. Among the patients, there were 1563 males and 441 females; there was a male-to-female ratio of 3.54:1 and an average age of (55.58 ± 12.77) years. A total of 1494 (74.55%) patients had hypertension, 98 patients had diabetes and impaired glucose tolerance (4.89%), 74 patients had coronary heart disease (3.69%), 11 patients had COPD (0.55%), 846 patients had a smoking history (42.2%), and 597 patients had a drinking history (29.8%).

### Distribution characteristics of the onset time of AAD

The circular distribution method showed that the onset time of AAD had a centralized trend (X = 0.1454, Y = 0.0350, *r* = 0.1495, α = 13.5446 °, and the number of days converted was 13.73 d). The theoretical peak day for new cases of aortic dissection was from January 13 to January 14. The homogeneity test confirmed that there was a clear concentration trend of AAD (Z = 44.808, *P* < 0.001).

### Comparison of daily meteorological conditions between “days with AAD” and “days without AAD”

The average minimum temperature, average maximum temperature, and average daily temperature differences in the “days with AAD” were lower than those in the “days without AAD” (all *P* < 0.05). According to the statistics from January 2016 to November 2019, it could be shown that there was no significant difference in the average air quality between the “days with AAD” and “days without AAD” groups. (Tables 1 and 2).

**Table 1:** The average minimum temperature, average maximum temperature, and average daily temperature difference in the “days with AAD” were lower than those in the “days without AAD”

**Group**	**N**	**Average minimum temperature** **[°C,M (Q**_**L**_,**Q**_**U**_**)]**	**Average maximum temperature** **[°C,M (Q**_**L**_,**Q**_**U**_**)]**	**Average daily temperature difference** **[°C,M (Q**_**L**_,**Q**_**U**_**)]**
Days with AAD	1333	17.00 (11.00,24.00)	25.00 (18.00,31.00)	7.00 (5.00,9.00)
Days without AAD	1185	20.00 (13.00,25.00)	27.00 (21.00,33.00)	8.00 (6.00,9.00)
Z		−6.914	−6.164	−3.600
P		0.000	0.000	0.000

**Table 2:** There was no statistical significance in the average air quality between “days with AAD” and “days without AAD” groups

**Group**	N	**Average air quality [°C,M (Q**_**L**_,**Q**_**U**_**)]**
**days with AAD**	**861**	**45.00 (35.00,57.00)**
**days without AAD**	**562**	**45.00 (35.00.57.00)**
**Z**		**−0.001**
**P**		**0.999**

**Fig. 1:**
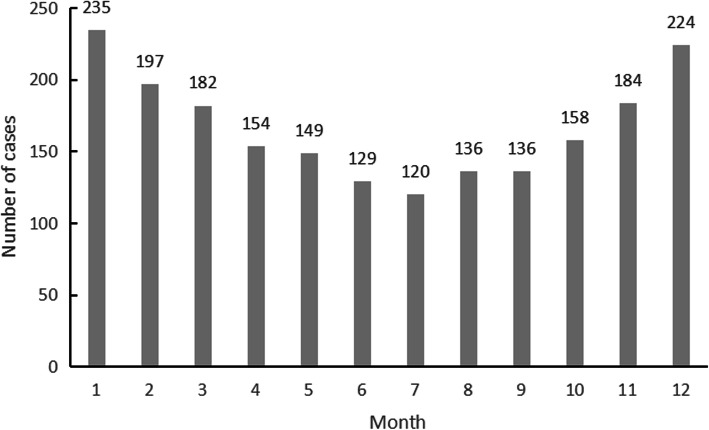
The number of AAD cases in each month

**Fig. 2:**
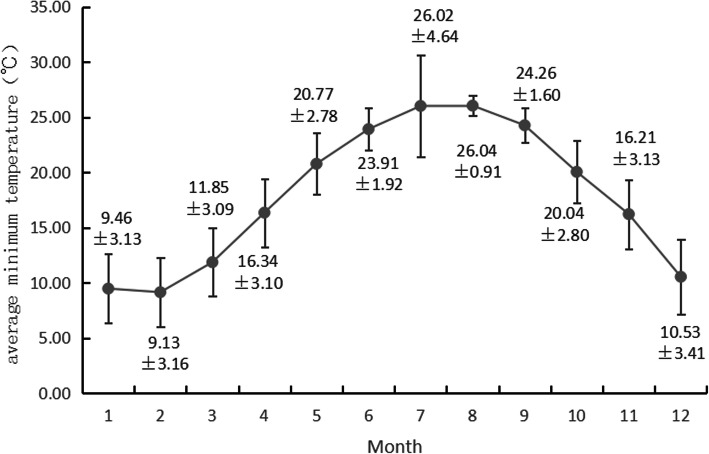
Average minimum temperature in each month

**Fig. 3:**
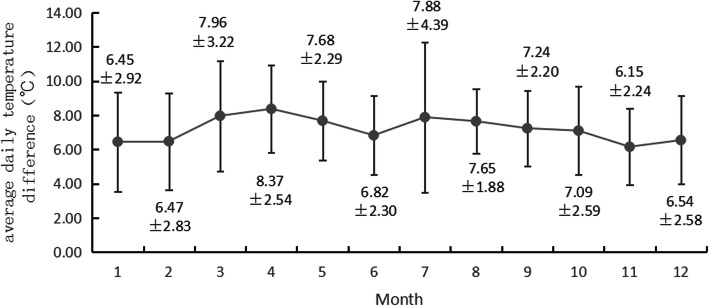
Average daily temperature difference in each month

The number of AAD cases, average minimum temperature, and average daily temperature differences in each month are shown in Figs. 1, 2 and 3. According to the characteristics of climate change, the four seasons of Fujian Province were divided as follows: March to May was spring, June to August was summer, September to November was autumn, and December to February was winter. From January 2013 to November 2019, the number of AAD cases was analyzed. The number of cases was the highest in January, followed by December, and the number of cases in June, July, August and September was lower than that in the rest of the months. The temperature distribution showed that February had the lowest average minimum temperature, followed by January; August had the highest average minimum temperature, followed by July; April had the highest average daily temperature difference, followed by March; and November had the lowest average daily temperature difference, followed by January.

The incidence of AAD in different average minimum temperature intervals and the average daily temperature differences are shown in Tables 3 to 4. The quartiles of the average minimum temperature were divided into < 11 °C, 11 ~ 16 °C, 16 ~ 23 °C and > 23 °C. The chi-square test was used to compare the differences in the incidence of patients with different sexes, ages, dissection types, and underlying diseases (including hypertension, coronary heart disease, diabetes, and COPD). The number of patients with AAD in the lowest average minimum temperature group (< 11 °C) did not show an increasing trend, and there was no significant difference in the number of patients with AAD in the different interval groups. The average minimum temperature was not found to be associated with the onset of AAD in different types A and B. Among patients of different ages, the number of patients with AAD in each group did not show an increasing trend with the decrease in the average minimum temperature, which may be related to the short duration of the average minimum temperature in Fuzhou, and more studies may be needed to determine the relationship; however, the total number of AAD cases was found to be largest in the 45–59-year-old age group in this study, followed by the 60–74-year-old age group, which is consistent with the results of many previous studies. With the increase in the average minimum temperature, the number of female patients gradually decreased, while the number of male patients was not significantly changed, suggesting that the incidence of female patients was more closely related to the change in the average minimum temperature. In addition, with the decrease in the average minimum temperature, the number of patients with hypertension gradually increased. Due to the small number of patients with coronary heart disease, diabetes, and COPD, it was not possible to observe the effect of different average minimum temperatures on the incidence of AAD with such diseases. Compared with daily temperature differences, no differences were found in the incidence of AAD in patients of different sexes, ages, dissection types, and underlying diseases (including hypertension, coronary heart disease, diabetes, and COPD).

**Table 3:** The incidence of AAD in different average minimum temperature intervals with different gender, age, type and underlying diseases (including hypertension, coronary heart disease, diabetes, COPD)

Average minimum temperature	Gender		Age (year)				Different type		Hypertension		Coronary heart disease		Diabetes		COPD	
	Male	Female	18–44	45–59	60–74	75–89	Type A	Type B	Y	N	Y	N	Y	N	Y	N
< 11.00	349	130	98	184	172	25	277	202	396	83	17	462	29	450	5	474
[11.00 ~ 16.00]	412	118	107	220	165	37	320	210	376	154	12	518	15	515	2	528
(16.00 ~ 23.00]	405	106	113	184	176	38	281	230	375	136	26	485	28	483	3	508
> 23.00	397	87	95	194	158	36	282	202	347	137	19	465	26	458	1	483
Total	1563	44	413	782	671	13	1160	84	1494	510	74	1930	98	1906	11	1993
χ^2^	12.432	1	7.391			6	3.136	4	22.735		5.941		6.854		3.073	
P	0.006		0.596				0.371		0.000		0.115		0.077		0.329

**Table 4:** The incidence of AAD in different average daily temperature difference intervals with different gender, age, type and underlying diseases (including hypertension, coronary heart disease, diabetes, COPD)

ADTD	Gender		Age (year)				Different type		Hypertension		CHD		Diabetes		COPD	
	Male	Female	18–44	45–59	60–74	75–89	Type A	Type B	Y	N	Y	N	Y	N	Y	N
< 5.00	293	69	73	149	114	26	209	153	263	99	10	352	15	347	2	360
[5.00,7.00]	562	159	147	270	258	45	431	290	548	173	34	687	35	686	1	720
(7.00,9.00]	430	113	121	203	174	45	306	237	402	141	21	522	28	515	5	538
> 9.00	278	100	72	160	125	20	214	164	281	97	9	369	20	358	3	375
Total	1563	44	413	782	671	1	1160	84	1494	510	74	1930	98	1906	11	19
χ^2^	6.642	1	8.921			36	1.836	4	1.578		4.878		0.649		4.516	93
P	0.084		0.445				0.607		0.664		0.181		0.885		0.175	

*ADTD* Average daily temperature difference

Changes in the average maximum temperature, average minimum temperature and average daily temperature from 5 days before the onset of AAD to the day of AAD onset are shown in Table 5. From 5 days to 2 days before the onset of AAD, the average maximum temperature and average minimum temperature showed a downward trend. From 5 days to 3 days before the onset of AAD, the average daily temperature difference also showed a downward trend. However, the statistical analysis results showed that there was no statistically significant difference in the average minimum temperature, average maximum temperature, or average daily temperature from 5 days before the onset of AAD to the day of AAD onset.

**Table 5:** Changes of the average maximum temperature, average minimum temperature and average daily temperature difference from 5 days before the onset of AAD to the day of AAD onset

Day before the day of AAD onset	N	Average maximum temperature (°C,^**−**^x ± s)	Average minimum temperature (°C, ^**−**^x ± s)	Average daily temperature difference (°C, ^**−**^x ± s)
5	1333	24.58 ± 7.55	17.31 ± 6.90	7.27 ± 3.05
4	1333	24.49 ± 7.55	17.29 ± 6.89	7.21 ± 2.97
3	1333	24.39 ± 7.55	17.25 ± 6.91	7.14 ± 2.94
2	1333	24.32 ± 7.59	17.16 ± 6.80	7.16 ± 2.85
1	1333	24.35 ± 7.53	17.17 ± 6.91	7.18 ± 2.99
0	1333	24.36 ± 7.54	17.21 ± 6.92	7.16 ± 2.84

## Discussion

AAD is not a rare cardiovascular emergency. Howard et al. conducted a survey in Oxfordshire, England, from 2002 to 2012. The results showed that the incidence of AAD was 4–7 per 100,000, and the prehospital mortality rate was as high as 18%. A total of 47.4% of patients with Stanford A aortic dissection and 13.3% of patients with Stanford B aortic dissection died within 30 days [13]. A survey of Chinese medical insurance research data showed that the hospitalization rate of AAD was 2/100,000, the estimated annual incidence of AAD was 2.8/100,000, and the overall patient mortality rate was 13.9% [14].

The clinical manifestations of AAD patients are complicated. Most patients die within hours to days after the onset of this disease. If appropriate treatment is administered immediately, the hidden danger of aortic rupture and the restoration of aortic blood supply can significantly reduce overall hospital mortality [15]. Therefore, studying the incidence of AAD, early detection and timely diagnosis are particularly important to reduce mortality in AAD patients. Mehta et al. analyzed international aortic registration study data and found that cold and temperate winters in the Northern Hemisphere were the peak periods of aortic dissection, and they believed that relative temperature might be a factor affecting the incidence of aortic dissection [16]. The seasonal volatility of aortic dissection has also been confirmed in related studies in many other countries [17–19].

We analyzed the onset time of 2004 patients with AAD in Fujian Province admitted to our hospital from 2013 to 2019. Aortic dissection was found to have a distinct seasonality. The winter was the peak season, and the number of cases was the lowest in summer. The number of cases was highest in January and lowest in July. The results of the study also suggested that the incidence of aortic dissection increased as the average minimum temperature, average maximum temperature and average daily temperature differences decreased, while the average air quality was not closely related to the occurrence of aortic dissection.

Guidelines for the diagnosis and treatment of aortic diseases issued by the European College of Cardiology recommend that all cases of suspected AAD should be predicted based on the condition, symptoms and clinical manifestations of the patients [20]. Studies on the correlation between aortic dissection and temperature need to be combined with studies of the characteristics of local meteorological conditions. For example, the results of Benouaich’s study only represented the relationship between the Mediterranean climate and the incidence of AAD in Toulouse [21]. Fujian is located on the southeastern coast of China in tropical and subtropical regions. The climate is a subtropical marine climate, with a high temperature, high humidity, long-term sunlight, and a large amount of solar radiation. The spring and autumn are short, and the winter and summer are long. The daily temperature difference is smaller than that in northern China. Exploring the impact of meteorological conditions on the incidence of AAD in the coastal areas of southeastern China is also of great significance for the early prediction and early diagnosis of AAD in such regions.

This study analyzed the distribution trend of AAD by using the circular distribution statistical method and obtained the following results. The incidence of AAD had a peak period, and the peak period was from January 13 to 14 each year. The distribution of AAD incidence in different months showed that January had the peak incidence of AAD, followed by December. This study indicated that February had the lowest average minimum temperature, followed by January. Traditional Chinese Spring Festival was in February, all Chinese people would have a long term holiday and there was a lot of population mobility. For example,the city where our hospital located would lose three-quarters of its population over this period. According to the traditional Chinese concept, during this period, patients’ willingness to seek medical treatment was low, and due to the influence of traffic and other factors, the rate of patients visiting provincial cities was low, while visiting the local hospitals was high. Maybe the actual incidence rate of AAD in February was higher, but that didn’t show up in our hospital records. The month with the highest incidence of AAD was basically consistent with the temperature trough, which was similar to Mehta and his team’s finding [16]. In cold months, people were prone to respiratory infections. As a cause of cardiovascular events, respiratory infections increased the incidence of cardiovascular events, including AAD, especially in patients with basic cardiovascular diseases. In addition, it was easier to consume a high-salt and high-fat diet in winter, while at the same time drinking more, which increased the possibility of cardiovascular disease. The results also suggested that the average minimum temperature, average maximum temperature and average daily temperature differences in the “with AAD day” were lower than those in the “without AAD day”, which proved that AAD was more likely to occur when the average temperature was low.

The results regarding the incidence of AAD in different temperature ranges indicated that the number of AAD cases in the lowest average minimum temperature group was not the largest, and the number of AAD cases in each group did not show an increasing trend with the decrease in average minimum temperature, which seemed to be contrary to Benouaich’s and other studies’ conclusions that the decrease in atmospheric temperature was the cause of AAD [21]. This might be due to the subtropical and tropical marine climate in Fujian Province, where the base temperature was higher than that of the Mediterranean climate studied by Benouaich et al. However, it could be found that with the decrease in the average minimum temperature, the number of AAD patients with hypertension increased gradually. Hypertension, as the most important susceptibility factor for AAD, might be related to the distribution of AAD in different months [22, 23]. The systolic and diastolic blood pressures of hypertensive patients were negatively correlated with ambient temperature [24]. Previous research has confirmed that low temperature is an important stressor of hypertension, leading to higher blood pressure in winter than in summer, and this trend is related to the decrease in winter temperature [25, 26]. In winter, the temperature is low, the sympathetic nervous system of the human body is activated, and catecholamine secretion is increased to cope with low temperature, which leads to increased heart rate and peripheral vascular resistance, resulting in increased blood pressure. The cold environment promotes the occurrence of high blood pressure, and by increasing blood friction against the vascular wall and surface shear stress, high blood pressure increases the risk of occurrence and rupture of aortic dissection in patients with a history of hypertension [21].

Benouaich et al. confirmed that the average temperature, maximum temperature, and minimum temperature from 3 days before the onset of AAD to the day of AAD onset decreased significantly, and the daily temperature differences were statistically significant, suggesting that dramatic changes in temperature might have an impact on the incidence of AAD [21]. This study analyzed the effects of average minimum temperature, average maximum temperature and average daily temperature differences on the onset of AAD over several days. The results showed that the average maximum temperature and average minimum temperature showed a downward trend from 5 days to 2 days before the onset of AAD, and the average daily temperature difference showed a downward trend from 5 days to 3 days before the onset of AAD. The decrease of temperature might have some influence on the occurrence of AAD. However, there was no significant difference in the average minimum temperature, average maximum temperature, or average daily temperature difference from 5 days before the onset of AAD to the day of AAD onset, which suggested that there was no clear correlation between daily temperature changes and the onset of AAD.

Most cardiovascular diseases, such as supraventricular tachycardia, myocardial infarction, ruptured abdominal aortic aneurysm, and subarachnoid hemorrhage, also have a similar seasonality of incidence as aortic dissection [27–30]. Low temperature might be used to increase the likelihood of AAD in patients with cardiovascular disease. Cold weather and high blood pressure are related to the incidence of AAD, which might originate from primary diseases such as hypertension, and atherosclerosis might also worsen due to low temperature. Therefore, for patients with high risk factors of aortic dissection, more attention should be paid to keeping warm and controlling hypertension during seasonal changes to reduce the risk of AAD.

This study was a single-center retrospective study with a limited sample size, different regions, different climatic conditions, different ethnic groups and other factors might lead to different conclusions. Moreover, there was a certain data selection bias, and some data might be missing due to the failure to cover out-of-hospital deaths. The meteorological bureau of our province could not provide the daily air quality data from January 2013 to December 2015. We excluded some aorto-related underlying diseases in the study, which might also have influenced the results. In the future, it would be possible to study the relationship between climate and patients with these diseases. The conclusions of the study still need to be further confirmed by large-scale multicenter studies. We also hope to accumulate more comprehensive registration data for patients with AAD in the future and to further explore the relationship between the incidence of AAD and climate change.

## Conclusion

The incidence of AAD had a clear, centralized distribution pattern, with the peak incidence occurring from January 13 to 14 each year. The peak incidence period coincided with the trough of temperature, and hypertensive patients with uncontrolled blood pressure at low temperatures are at risk for AAD.

## Data Availability

Data sharing not applicable to this article as no data sets were generated or analyzed during the current study.

## Abbreviation

AAD: Acute aortic dissection
